# Incorporating multiple-marker information to detect risk loci for rheumatoid arthritis

**DOI:** 10.1186/1753-6561-3-s7-s28

**Published:** 2009-12-15

**Authors:** Xuexia Wang, Huaizhen Qin, Qiuying Sha

**Affiliations:** 1 Department of Mathematical Sciences, Michigan Technological University, Houghton, Michigan 49931, USA; 2 Capital University of Economics and Business, Beijing 100026, PR China

## Abstract

In genome-wide association studies, new schemes are needed to incorporate multiple-locus information. In this article, we proposed a two-stage sliding-window approach to detect associations between a disease and multiple genetic polymorphisms. In the proposed approach, we measured the genetic association between a disease and a single-nucleotide polymorphism window by the newly developed likelihood ratio test-principal components statistic, and performed a sliding-window technique to detect disease susceptibility windows. We split the whole sample into two sub-samples, each of which contained a portion of cases and controls. In the first stage, we selected the top *R *windows by the statistics based on the first sub-sample, and in the second stage, we claimed significant windows by false-discovery rate correction on the *p*-values of the statistics based on the second sub-sample. By applying the new approach to the Genetic Analysis Workshop 16 Problem 1 data set, we detected 212 out of 531,601 windows to be responsible for rheumatoid arthritis. Except for chromosomes 4 and 18, each of the other 20 autosomes was found to harbor risk windows. Our results supported the findings of some rheumatoid arthritis susceptibility genes identified in the literature. In addition, we identified several new single-nucleotide polymorphism windows for follow-up studies.

## Background

Rheumatoid arthritis (RA) is a common chronic destructive disease of an unknown complex etiology. Both genetic and environmental bases are thought to contribute to this disease. The human leukocyte antigen (HLA) region major histocompatibility complex (MHC) on chromosome 6 (6p21.3) is known to be associated with RA. This region is the only one that has been consistently shown to be both linked and associated with RA across all populations. It extends over 3.6 Mb and is divided into three sub-regions (classes I, II, and III). It is a highly dense area containing about 220 genes, many of which are thought to have immunoregulatory functions [1]. Recently, matured genotyping technology and availability of large case-control collections have made it possible to detect mild risk loci. The Genetic Analysis Workshop 16 (GAW16) Problem 1 data set is such a large scale case-control study which contains genotypes at 531,689 single-nucleotide polymorphisms (SNPs) on chromosomes 1-22 for 868 cases and 1194 controls.

Recently, many approaches, e.g., Hotelling's *T*^2 ^test [2,3] and the linkage disequilibrium (LD) contrast tests [4,5], have been proposed to detect multiple-marker association. The Hotelling's *T*^2 ^test and the LD contrast test compare the means and the variance-covariance matrices of genotype scores between cases and controls, respectively. More recently, we proposed a likelihood ratio test-principal component (LRT_PC) to compare the means and the variance-covariance matrices of genotype scores simultaneously [6]. However, all of these approaches only allow a SNP region of several to tens of markers.

In this article, we report a novel genome-wide sliding-window approach to detect genetic association between a trait and SNP regions. This approach integrated the LRT_PC with the concept of sliding window [7] and the basic idea of two-stage approaches [8]. Applied to the GAW16 Problem 1 data set, our approach yielded results that support the findings in the literature of some RA susceptibility genes on chromosomes 1, 2, and 6 and detected more SNP windows for follow-up studies.

## Methods

### LRT_PC statistic

We recently proposed a LRT_PC approach to test the association between a given SNP window and a disease status [6]. To calculate the test statistic, we first perform principal component (PC) analysis to the genotype scores of the sampled individuals. Then, the LRT_PC test statistic is given by , where *n *and *m *are the numbers of cases and controls, respectively, and the  values are the sample variance-covariance matrices of the first *K *PCs in cases, controls, and the pooled sample, respectively. Wang et al. [6] showed that the LRT_PC test is more powerful than the Hotelling's *T*^2 ^test and the LD contrast test [2-5] in most cases. The power of the LRT_PC test is perhaps due to its ability to capture the differences of the means and the variance-covariance matrices of genotype scores in cases and controls simultaneously.

### Two-stage sliding-window approach

Because the LRT_PC test may be more powerful than other multi-marker tests, we wanted to use it to analyze the data set of GAW16 Problem 1. However, the LRT_PC can only be applied to a small chromosome region. To apply the LRT_PC to genome-wide association studies, we propose a sliding-window approach [7]. To use sliding windows, we divide all SNPs into contiguous overlapping windows and apply the LRT_PC in each window. Suppose that we use windows with a window size of *S*, then, all the SNPs can be divided into windows 1 to *S*, 2 to *S *+ 1, 3 to *S *+ 2, and so on.

Because we do not know the distribution or asymptotic distribution of the test statistic LRT_PC, we need to use a permutation approach to estimate the *p*-value of the test. For a genome-wide association study, the number of windows usually is more than 500,000 and the number of permutations usually is no less than 1000 (100,000 permutations were used in this study). The computation is not feasible for the sliding-window approach discussed above. Thus, we propose a two-stage approach. In the two-stage approach, we split all individuals into two sub-samples. In the first stage, by assuming that all individuals are genotyped at all SNPs, we use the first sub-sample to select *R *most promising SNP windows with the largest values of the LRT_PC statistic calculated via the first sub-sample. In the second stage, only the genotypes at SNPs within the *R *most promising windows are used. In this stage, we use the second sub-sample to assess *P *values for the *R *selected windows by permutations and claim significance by the false-discovery rate (FDR) correction in Benjamini and Hochberg [9]. For the two-stage approach, we only need to do permutations in the second stage. Thus, the two-stage approach is computationally much more efficient than one-stage approach.

To analyze the data set of GAW16 Problem 1 using LRT_PC based two-stage sliding-window approach, we use the following settings: window size is 5; the number of windows selected in the first-stage, *R*, is 1000; the sample size of the first sub-sample is 15% of the total sample (15% cases and 15% controls). In the first stage, the number of PCs used in the LRT_PC test in each window is 5, i.e., we do not perform PC analysis. In the second-stage, the number of PCs used in the LRT_PC test in each window, *K*, is decided by the fact that the first *K *PCs can explain 85% of the total variability.

To choose the sample size of the first sub-sample, we did a power analysis based on a single-marker test similar to that of Wang et al. [8]. Our results showed that the optimal value of the sample size of the first sub-sample is between 10% and 30% of the total sample. We use the results based on a single-marker test as a reference to choose the sample size of the first sub-sample in this study (15% of the total sample).

As pointed by Skol et al. [10], our proposed two-stage approach may be not as powerful as joint analysis. However, the results of Skol et al. also showed that when the sample size of the first sub-sample is small (15% of the total sample), the power difference between the two-stage approach and joint analysis is also small. To compare the power of the two-stage and one-stage approaches, we have done a small scale simulation study (10,000 SNPs and 1000 permutations). The simulation results showed that when the first sub-sample is 15% of the total sample, the power difference between the two approaches is also small. In summary, compared with the joint analysis and one-stage approach, our proposed two-stage approach has a small power loss in exchange for a big increase in computational efficiency.

## Results

We applied the proposed two-stage sliding-window approach to analyze the RA data set from GAW16 Problem 1. After removing duplicated and contaminated samples, this data set contained genotypes at 531,689 SNPs on chromosomes 1-22 for 868 cases and 1194 controls. The missing genotypes were imputed using the method of Browning and Browning [11]. First, we used the first sub-sample to calculate the LRT_PC statistic for each of the 531,601 windows and selected 1000 of the most promising windows with the largest values of the LRT_PC test statistic. Then, we used the second sub-sample to calculate the LRT_PC test statistic for each of the 1,000 selected windows and used 100,000 permutations to evaluate the *p*-values. In total, we discovered 212 significant windows by the FDR correction at a nominal level 0.05, among which 126 windows are on chromosome 6. Many of the 212 windows are overlapped, especially for the windows on chromosome 6. After merging the overlapped windows, there were 68 non-overlapped windows left. Among the 68 windows, 26 of them were on chromosome 6. The 26 windows on chromosome 6 were in a region (28,292,350 to 33,349,147 bp) in high LD with HLA-DRB1, a factor known to have a strong association with RA. The details of these non-overlapped windows (except those on chromosome 6) are summarized in Table 1.

**Table 1:** The details of non-overlapped significant windows

Window ID	Chr	Physical Location	Genes	References
1	1	792429, 1071463	*FAM87B, C1orf159*	
2	1	149769454, 149786537	*FCRL3*	[13]
3	2	139347733, 139370993	*NXPH2*	
4	2	158103376, 158124755	*CYTIP*	
5	2	192062916, 192117323	*MYO1B*	
6	2	193087360, 193117091	*STAT4*	[12]
7	2	204487030, 204527849		
8	2	217410540, 217413523		
9	3	61980695, 61998337	*PTPRG*	
10	3	112000554, 112031324		
11	5	25934777, 25967191		
12	5	111055309, 111062116		
13	5	137614229, 137669929	*GFRA3*	
14	5	175278829, 175508504		
15	7	35309745, 35321578		
16	8	20380853, 20411350		
17	9	34654488, 34675940	*CCL27*	
18	9	84179401, 84220779	*SLC28A3*	
19	9	91845119, 91920813		
20	10	5016096, 5053944		
21	10	49685217, 49698112	*WDFY4*	
22	10	87994785, 88004329	*GRID1*	
23	11	67868248, 67886182	*LRP5*	
24	11	68807443, 68825321		
25	12	130500249, 130524477	*hypothetical LOC116437*	
26	13	73411181, 73419864	*KLF12*	
27	13	113652806, 113781019	*FAM70B*	
28	14	31921536, 31942503	*AKAP6*	
29	14	80898925, 80930993	*STON2*	
30	15	72678184, 72721493		
31	16	2452524, 2582219	*C16orf59*	
32	16	9214168, 9232588		
33	16	12651732, 12676977		
34	16	64715655, 64730155		
34	16	64715655, 64730155		
35	16	64735494, 64764278		
36	16	67451454, 67514126	*TMCO7*	
37	17	34194598, 34251205	*PIP4K2B*	
38	17	68919134, 68930918	*SDK2*	
39	19	41254897, 41282169	*WDR62*	
40	22	24746347, 24757608	*MYO18B*	
41	22	24760841, 24782942		
42	22	43076969, 43084289		

Note: There are 26 significant non-overlapped windows on chromosome 6 which are in a region (28,292,350 to 33,349,147 bp) in high LD with HLA-DRB1, a factor known to have a strong association with RA. We did not list those 26 non-overlapped significant windows due to space limitations.

For validation purposes, we used the SNP Search Engine to find genes which contain or are near to SNPs that were discovered. We found rs2357135 nesting at "2q32", which is near to gene "*STAT 4*" and thus supported the finding in Remmers et al. [12]. In addition, our discoveries supported the findings of *FCRL3 *on chromosome 1 [13] and HLA region (MHC) on chromosome 6 [1]. Additionally, we detected many novel risk windows for follow-up studies. Except for chromosomes 4 and 18, each of the other autosomes was found to carry risk windows. For example, we detected SNP rs2047465 which nests in gene *SDK2 *on chromosome 17.

## Discussion

In this article, we proposed a two-stage sliding-window approach and, in each window, our recently proposed LRT_PC test was applied to test the association between a window and a disease. Then, we applied the method to GAW16 Problem 1 to detect risk windows for RA. Different existing RA association studies discovered diverse susceptibility genes on all chromosomes except for MHC candidate genes on chromosome 6. Our analysis supported the findings of some RA susceptibility genes that have been identified to be associated with RA in the literature.

It is intractable to formulate the null distribution of the LRT_PC statistic and thus the permutation approach must be applied to evaluate the *p*-values of top *R *promising windows (*R *= 1000 was used in this study). For a given *R*, the number of permutations must be large enough to obtain accurate *p*-values. Thus, the choice of *R *partially depends on computational capacity. Further efforts are needed to determine the optimal value of *R*.

In this study, we used 5 PCs, i.e., we did not perform PC analysis in LRT_PC test for each window of size 5 in the first stage. We did not perform PC analysis (or use 5 PCs) in the first stage for two reasons. One is that we have to use the same number of PCs in different windows so that the values of the test statistic in different windows are comparable. The other is that in the first stage, we only use the test statistic to rank the SNP windows and the results are similar when using different numbers of PCs (results not shown). Another remaining question regarding the proposed method is how to choose the window size. Most often, researchers use windows of size 3. In the LRT_PC test, we use PC analysis to reduce the dimension, and thus we can use a larger window size (i.e., window size of 5).

## Conclusion

In this article, we proposed a two-stage sliding-window approach to detect the association between SNP windows and a disease status. Application to GAW16 Problem 1 data set illustrated its practical advantages. Our results supported the findings of several genes which were identified to be responsible for RA in the literature. We also discovered several additional SNP windows for follow-up studies.

## List of abbreviations used

FDR: False-discovery rate; GAW16: Genetic Workshop Analysis 16; HLA: Human leukocyte antigen; LD: Linkage disequilibrium; LRT-PC: Likelihood ratio test-principal component; MHC: Major histocompatibility complex; PC: Principal component; RA: Rheumatoid arthritis; SNP: Single-nucleotide polymorphism

## Competing interests

The authors declare that they have no competing interests.

## Authors' contributions

XW performed the statistical analysis and wrote the first draft of the manuscript. HQ participated in the study design and helped to draft the manuscript. QS contributed to the design of the study and to the manuscript preparation. All authors read and approved the final manuscript.
